# New perspectives on analysing data from biological collections based on social network analytics

**DOI:** 10.1038/s41598-020-60134-y

**Published:** 2020-02-25

**Authors:** Pedro C. de Siracusa, Luiz M. R. Gadelha, Artur Ziviani

**Affiliations:** National Laboratory for Scientific Computing (LNCC), Petrópolis, RJ 25651-075 Brazil

**Keywords:** Network topology, Biodiversity, Scientific data

## Abstract

Biological collections have been historically regarded as fundamental sources of scientific information on biodiversity. They are commonly associated with a variety of biases, which must be characterized and mitigated before data can be consumed. In this work, we are motivated by taxonomic and collector biases, which can be understood as the effect of particular recording preferences of key collectors on shaping the overall taxonomic composition of biological collections they contribute to. In this context, we propose two network models as the first steps towards a network-based conceptual framework for understanding the formation of biological collections as a result of the composition of collectors’ interests and activities. Building upon the defined network models, we present a case study in which we use our models to explore the community of collectors and the taxonomic composition of the University of Brasília herbarium. We describe topological features of the networks and point out some of the most relevant collectors in the biological collection as well as their taxonomic groups of interest. We also investigate their collaborative behaviour while recording specimens. Finally, we discuss future perspectives for incorporating temporal and geographical dimensions to the models. Moreover, we indicate some possible investigation directions that could benefit from our approach based on social network analytics to model and analyse biological collections.

## Introduction

How data is classified in information infrastructures directly impacts our potential knowledge about different domains^1–3^. Indeed, biological collections stand as invaluable sources of primary biodiversity information, physically storing biological materials that testify to the existence of living organisms over time and geographic space. Regarded as important natural history repositories, biological collections have been increasingly used for a multitude of ecological and conservationist investigations^4–7^. As many of these initiatives require intensive use of biodiversity data, typically covering wide geographic areas and long periods of time, they would become impracticable without biological collections, due to the high costs associated with collecting new data in the field on demand. Besides, more species have been recently discovered by taxonomists by inspecting unidentified materials at biological collections than by exploring and collecting at new locations^6^. One important limitation, however, is that biological collections provide only a sampled partial view of the actual biological diversity within their actuation regions. Furthermore, applications aiming at investigating wider ecological and biogeographic processes should be able to combine data from multiple biological collections. In this work, we are particularly motivated by the challenge of characterizing *sampling biases* in data, defined as systematic errors that are introduced in data as an effect of not using random sampling designs^8^. Sampling biases are typically introduced in biodiversity datasets when collectors record specimens in the field in an opportunistic fashion, deploying uneven sampling efforts throughout the studied area and recording preferentially organisms with particular characteristics over others. In addition, collectors consider the accessibility of potential sampling sites while selecting them, and thus locations such as roadsides and the proximities of urban centers are often oversampled^8^, while others that are more remote remain poorly represented. Indeed, what is collected is typically a product of both targeted collecting (based on academic interest) and opportunistic collecting (based on ecological and phenotypic characteristics such as abundance, phenotypic distinctiveness, and so on). Sampling biases are in fact one of the main limitations of biological collections, and have been observed to strongly impact the overall quality of models in case they fail to account for them^9–11^. Characterizing bias in such datasets would therefore require a systematic analysis of how the complex arrangements of the perceptions, interests, and interactions of collectors shape the overall composition of the collections. Not surprisingly, a number of recent efforts has been made to quantify the extent of biases, in particular sampling completeness^12,13^.

Within this scope, we propose two network models as the first steps towards a network-based conceptual framework for understanding the formation of biological collections as a result of the composition of collectors’ interests and activities. This paper thus contributes towards a novel modeling approach, based on social network analysis, for investigating the assemblage of biological collections as a *social process*, resulting from the collecting activities of collectors and their collaborative interactions. Networks have been used in a wide range of domains for the investigation of complex systems of interacting entities, from studies of the World Wide Web^14^ to ecological interaction webs^15^. However, to the best of our knowledge, social network analysis has not yet been applied in biodiversity informatics for investigating the assemblage of biological collections. One recent study uses network analytics to investigate the connectivity and roles of many organizations in the biodiversity informatics landscape, in terms of how they exchange information^16^. Grounded on recent advances in network science theory^17–19^ and social network analytics^20,21^, in this work, we introduce two classes of *network models*: (*i*) Species-Collector Networks (SCNs) for analysing interest relations involving collectors and species; and (*ii*) Collector coWorking Networks (CWNs) for structuring collaborative relations involving pairs of collectors.

We demonstrate the practical use of our network models by carrying out a case study, using the species occurrence dataset from the University of Brasília Herbarium (UB), downloaded through the GBIF platform. Once the network models are built, we explore their basic topological features and investigate the formation of communities (interest communities in SCNs and coworking communities in CWNs). We also investigate the relative relevance of collectors in the herbarium, regarding both their taxonomic contributions and their social positions.

Finally, we believe our network models open new perspectives for research in biodiversity informatics, specifically for applications that rely on data from biological collections. With further developments from our work, we expect to provide a mechanism for systematically classifying collectors according to their expertises, their behaviours, and their social roles in the collections they contribute to. Another perspective is to enrich species occurrence datasets with contextual information, inferred by observing the composition of collectors associated with each record (and their respective profiles). Moreover, although we have not yet incorporated the temporal and geographical dimensions to the structure of our networks in this work, we believe this would be a fundamental advance, allowing to investigate how collectors interact and which species they record through time and geographic space.

## Methods

Network science refers to a relatively new domain of scientific investigation, which aims at describing emergent properties and patterns from complex systems of interacting entities^17–19^. Such relational systems are naturally represented as networks, in which interactions are represented as pairwise connections (*links*) between entities (*nodes*) and assume particular semantics depending on the nature of the modeled phenomenon. Next, we introduce and formally describe two classes of network models that were developed during this study.

### Species-Collector Networks (SCNs)

SCNs are a particular type of *interest networks* describing relationships of type “collector samples species” or, conversely, “species is sampled by collector”. The network is thus composed of collectors holding links to every single species they have ever recorded or, alternatively, species holding links to every collector who has ever recorded them. An important semantic aspect of this model worth emphasizing is that here we model collectors recording *species* rather than *specimens*. Each occurrence record that is used to build the network includes a single specimen, which is a representative of a species. Thus, while collectors are represented in the network at the individual level (each collector is a person), species are instead represented as entities comprising groups of individuals. Nevertheless, each species is uniquely represented as a node in the network.

As collectors and species refer to distinct entities in our system, we represent them in our network as two disjoint node sets. This matches the description of a bipartite network *SCN* = (*S*_*col*_, *S*_*sp*_, *E*),  where *S*_*col*_ = {*u*_1_, *u*_2_, . . . , *u*_*n*_} is the node set representing the collectors group; *S*_*sp*_ = {*v*_1_, *v*_2_, . . . , *v*_*m*_} is the node set representing the species group; and *E* is the set of undirected edges between members of *S*_*col*_ and *S*_*sp*_. The bipartite graph can also be represented as a rectangular biadjacency matrix *A*^*n*×*m*^ for which *a*_*ij*_ ≠ 0 *iff* (*u*_*i*_, *v*_*j*_) ∈ *E*.

A SCN model is built from a species occurrence dataset by using basically two fields. The first is the collectors field, containing the names of all collectors that were responsible for the record; and the second one is the species field, storing the species identity assigned to the specimen in the record. The network is built up from the dataset in an iterative process, in which weighted edges linking collectors to species are structured from rows in the dataset. For each new record containing *n* collectors, *n* links connecting each individual collector to the recorded species are created (or strengthened, in case they already exist). In the end of the process, the strength of each link is equivalent to the number of times each species-collector association appears in the original dataset. Link strength is graphically represented by the edge thickness. The strengh of each edge is stored in the biadjacency matrix A, and thus the value of element *a*_*ij*_ is the number of times the edge (*u*_*i*_, *v*_*j*_) occurs.

The entire set and counts of species a collector has recorded in a dataset, which can be thought as a collector’s species signature, composes his/her *species bag*. This attribute is therefore exclusively derivable for collector nodes and this is a convenient structure for comparing collectors in terms of the composition of their records, i.e. their respective species bags. For that task, a high variety of well-known distance algorithms for vectors in literature can be readily applied. The species bag ${\sigma }_{{u}_{i}}$ for collector *u*_*i*_ is thus defined as

1 $${\sigma }_{{u}_{i}}=\left[{a}_{i1},{a}_{i2},...,{a}_{im}\right],$$

where *m* is the length of the species set and each *a*_*ij*_ is the total number of records of species *v*_*j*_ by collector *u*_*i*_. The sum of all elements in a collector’s species bag corresponds to the total number of records for that collector *u*_*i*_.

The entire set and counts of collectors who have recorded a particular species in a dataset comprise its *quorum*, an exclusive attribute of species nodes. This concept can be thought as the inverse of a species bag, being the collector signature of a species. The quorum vector ${\iota }_{{v}_{j}}$ of a species *v*_*j*_ is directly obtained from the graph’s biadjacency matrix as the *j*^*th*^ column-vector, i.e.,

2 $${\iota }_{{v}_{j}}=[{a}_{1j},{a}_{2j},...,{a}_{nj}],$$

where *n* is the length of the set of collectors and each element *a*_*ij*_ is the total number of times collector *u*_*i*_ has recorded species *v*_*j*_. The total number of occurrences of species *v*_*j*_ in the entire dataset is the sum of all elements in its quorum vector.

In some contexts, it might be desired to simplify SCNs by grouping species nodes into higher taxonomic ranks (or levels), such as a *genus* or a *family*. This process is defined as a *taxonomic aggregation*, and is performed by (*i*) obtaining a grouping of species using some taxonomic rank; (*ii*) obtaining quorum vectors for each species; (*iii*) summing up quorum vectors for all species in each group; and (*iv*) building a new SCN model, aggregated on taxonomic rank *T*. The *taxonomic resolution* of a SCN is thus the taxonomic rank at which species are aggregated in the model. For the sake of model interpretability, all nodes in *S*_*sp*_ must necessarily be taxa belonging to the same taxonomic rank as the taxonomic resolution adopted for the model.

### Collector coWorking Networks (CWNs)

CWNs are a particular instance of *collaboration networks* describing co-authoring relationships between collectors from species occurrence records. We consider two collectors to be co-authors in a given record if they are both included in the collector field for that record. The collector field holds a list of the names of the collectors who have authored each record in the dataset. We refer to each distinct list of collectors in this context as a team. As opposed to SCNs, which basically describe the interests of collectors towards species, relationships in CWNs are directly formed between collectors who have effectively worked collaboratively in the field, i.e. the coworking (team) behaviour around the registering of one species record is reflected as a group of fully connected collectors in a CWN. These collaborative relationships are semantically described as “collector records specimen with collector”. Each individual species occurrence record with at least two collectors (i.e., team size greater than 1) is thus considered a distinct collaboration act, originating new pairwise connections between all the involved collectors. For records with team size equal to 1, which we refer to as non-collaborative records, no connections are created. Differently from SCNs, where two classes of entities are represented in the graph as disjoint node sets with the bipartite constraint, CWNs exclusively model direct relationships between entities from a single class (collectors), with the only connectivity restriction that a collector should not hold collaborative ties to itself. The model is thus formally described as an undirected graph *CWN* = (*S*, *E*),  where *S* = {*u*_1_, *u*_2_, . . . , *u*_*n*_} is the graph’s node set of collectors and *E* is the set of undirected edges linking members of *S*.

Weights are assigned to edges in the CWN as a measure of their overall relevance in the network structure. Edges with higher weight values represent stronger collaborative ties between collectors, pointing out the main groups of collectors who are most willing to collaborate. However, as pointed out by other authors studying social networks^22^, in reality not all collaboration acts should contribute in the same way for a collector’s network. Collectors tend to hold weaker collaborative ties with each other when they collaborate in larger teams than when they collaborate in smaller teams. Therefore, we use a hyperbolic weighting rule to account for this fact, while also considering the total number of collaborations between two collectors as a factor contributing to the strength of their common link. According to this rule, not every new occurrence of the link increases the edge weight equally. The contribution of each new link depends on the number of the collectors *n*^(*k*)^ included in record *k* or, in other words, the team size. This rule follows a hyperbolic growth function

3 $${w}_{(i,j)}=\sum _{k}\frac{{\delta }_{i}^{(k)}{\delta }_{j}^{(k)}}{({n}^{(k)}-1)},$$

where ${\delta }_{u}^{(k)}=1$ if collector *u* is in record *k*, and 0 otherwise. As the hyperbolic function in Eq. 3 has singularity at 1, it gets ill-defined for records with only one collector. Therefore, in this case, only records with two or more collectors are used to compute edge weights. The maximum weight contribution of 1 is assigned to records with two collectors, whereas records with larger teams of collectors yield smaller individual contributions.

Relationships in the CWN graph can be represented in a symmetric adjacency matrix *A*^*n*×*n*^ for which *a*_*ij*_ ≠ 0*iff* (*u*_*i*_, *u*_*j*_) ∈ *E*. Values of non-zero elements depend on the weighting method adopted for representing link strength, e.g. simple full count or hyperbolic weighting for fractional counting. Additionally, the model’s connectivity constraint states that all diagonal elements in *A* are necessarily equal to 0, thus ensuring that no self-loops are formed.

Although structurally distinct, SCNs and CWNs provide complementary perspectives on the recording behaviour of collectors from a given species occurrence dataset. From SCNs, we retrieve information on which collectors have recorded which species and, conversely, which species were recorded by which collectors. On the other hand, CWNs allow us to investigate which collectors team up with whom during fieldwork.

## Results

In this section, we use the network models proposed in the Methods section to understand aspects regarding the taxonomic preferences and the collecting behaviour of collectors who have contributed to the University of Brasília (UB) Herbarium with specimens records.

### Data

In this case study, we have used the entire digitized collection of records from the UB Herbarium^23^, which is publicly available for download through the Global Biodiversity Information Facility (GBIF) data portal^24^. We have used the *Python v.3.6* language loaded with packages *Pandas*, *Numpy*, and *Matplotlib* for exploring the occurrence dataset; and the *Caryocar* package (designed and implemented by us, in the context of this work) for programatically constructing the SCN and CWN models from occurrence data. At the time of this study, the entire occurrences dataset from the UB herbarium had a total of 185,311 records and 235 fields, covering records from 1800 to 2017, although approximatelly half of the records are from the last 30 years (1988–2017). For our application, however, only a small subset of those fields were considered to be relevant and were thus included in our analysis: *recordedBy*, *eventDate*, *stateProvince*, *countryCode*, *decimalLatitude*, *decimalLongitude*, *issue*, *scientificName*, and *taxonRank*. Most of these fields (except for *issue*, which describes data quality issues found in the dataset) follow *Darwin Core* terms standards^25^.

Before we could use the UB Herbarium dataset for actually building the network models, we submitted the tabular dataset to some data filtering and transformation routines. The data preparation process consisted of (*i*) selecting occurrence records from which relevant social ties could be derived for both network models; (*ii*) extracting atomized collector names from the *recordedBy* field, which originally contains a string of names; (*iii*) normalizing the extracted collector names to obtain their id’s; (*iv*) resolving inconsistencies on collector names and mapping name variants to entities; and (*v*) filtering out inadequate collector names. All this data pre-processing, data cleaning routines, and data transformation is done by the code available in the *Caryocar* package. All the presented analyses used the dataset from Munhoz *et al*.^23^ and our package (examples on how to do it are provided with the package as well). The result is the SCN and CWN models we further discuss in the remainder of this section. In the presented figures, for a richer visualisation experience^26^, we also add links to browsable interfaces that reveal the scale and complexity of the represented networks.

### The UB Species-Collector Network

The UB SCN model has a total of 6,768 collectors and 15,344 species nodes, with a total of 142,647 undirected edges connecting nodes from opposite sets.

#### Connected components

The UB SCN is composed of a total 351 connected components, the largest of which (the giant component, or *c*_1_) contains the majority of nodes in the network (93.6% of the collectors and 95.0% of the species). From a collector’s perspective, the requirement for it to belong to the giant component is that it must have collected at least one species in common with another collector who is already included in the giant component. The same reasoning applies to species nodes, by observing the inverse relationship. Apart from the giant component, most other connected components contain as few as two or three nodes, representing collectors who have never recorded a species in common with any collector from *c*_1_; and conversely, species that have never been collected by any of those collectors that belong to *c*_1_. One of those 350 remaining connected components, however, is considerably larger than the others, with a total of 3 collectors and 141 species. We refer to it as the second largest component (*c*_2_). Further, *c*_1_ is mostly composed of species from phylum *Tracheophyta* (88% of all records), followed by phylum *Bryophyta* (mosses), comprising 8% of the records. Component *c*_2_, on the other hand, is represented by algae (phyla *Charophyta*, *Chlorophyta*, comprising 91.6% of all records), bacteria (phylum *Cyanobacteria* (4.3%)), and other microscopic eukariotic organisms (phyla *Euglenozoa*, *Myzozoa* (4.1%)), which are taxonomically distinct from the vast majority of species in the herbarium. The remaining components (*c*_3_, *c*_4_, . . . , *c*_351_) include a total of 431 distinct collectors and 446 distinct species.

#### Communities of common interests

Communities in SCNs are formed by groups of collectors who are more interested towards particular subsets of species than are other collectors, external to the group. As the number of edges linking members of a community with other members tends to be larger than those connecting members to non-members, communities can be visually detected as distinguished clusters of nodes in the network when using force-directed algorithms^27^ for graph layout, thus no community detection algoritm was needed.

**Figure 1:**
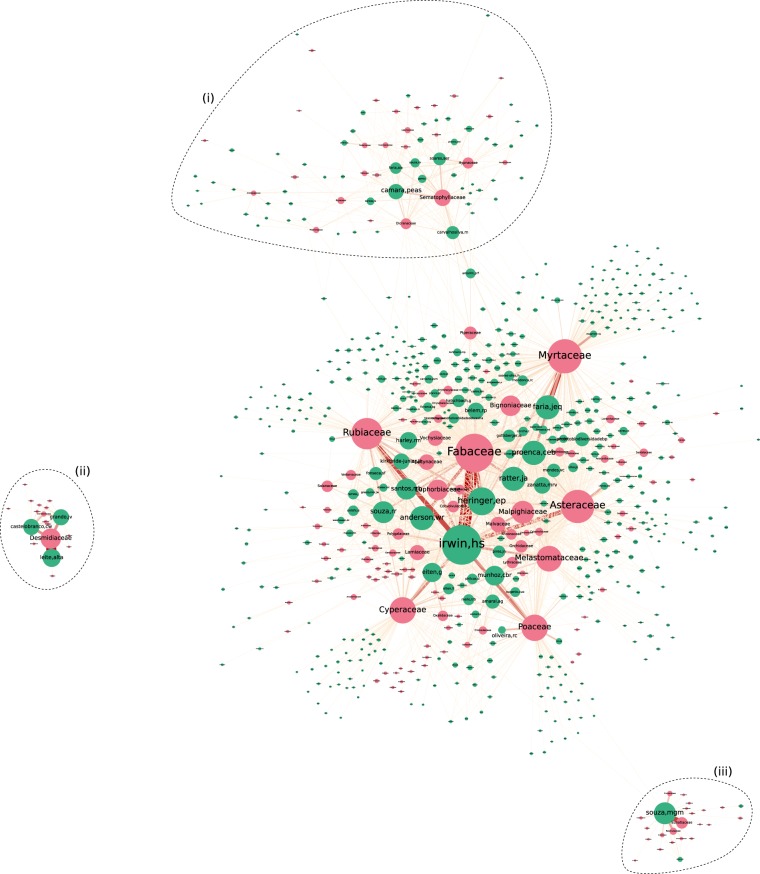
General aspect of the UB SCN, taxonomically aggregated at the family rank. Species and collector nodes are colored in pink and green, respectively. Node size is proportional to how often a collector or specimen appears on records, whilst the width of edges reflect their weight. Polygons (i), (ii), and (iii) are placed around communities that are visually most distinguishable. This figure has been originally presented in the first author’s MSc thesis^28^. For a better visualization experience refer to the interactive version of the graph (https://lncc-netsci.github.io/pedrocs/networks/ub_scn).

As the size of the UB SCN is relatively large for it to be informative in a static figure, we summarized the network in two steps. First we aggregated the SCN onto the *family* rank, as it would be impractical to draw relevant conclusions from the network if every single species were plotted in the figure. By performing the taxonomic aggregation, we reduced the number of *S*_*sp*_ nodes (taxa) from 15,344 to 474, although the number of edges (from 142,647 to 43,803) did not decrease in the same proportion. This incurred in a 10 ×  increase in network density, from 1.37 × 10^−3^ to 1.36 × 10^−2^. In the second step of the summarization routine, we removed collector-family ties that occurred less than 20 times throughout the entire dataset. As this edge filtering routine resulted in many isolated collectors, most of which novice collectors with low absolute recording counts, we also omitted them as to improve the figure readability. Three communities are visually distinguishable from the central region of the network, which we refer to as the network core (Fig. 1). Although the network core could be considered a community *per se*, we prefer to think of it as a region that best reflects the overall interests of the majority of the collectors contributing to the herbarium. Nevertheless, collectors from the network core still vary considerably regarding their recording interests, as it can be verified by inspecting the sets of taxa they’re linked to and the strength of their connections. Those who have sampled organisms from many distinct families (and are thus considered to display a more generalist collecting behaviour) are placed more centrally in the network core by the graph layout algorithm, whereas those who are more specialists are consequently pushed towards the borders of the network core, as near as possible to their most recorded taxa.

Represented as the biggest hub in the network, *Howard S. Irwin* (*irwin,hs*) is the collector with most records in the network, having intensively collected organisms from many distinct families, especially from the most central ones (illustrated in Fig. 1 as the largest pink nodes in the network core). The majority of his records are, in descending order, from families *Fabaceae*, *Rubiaceae*, *Asteraceae*, *Poaceae*, and *Cyperaceae*. He is also the collector holding the highest number of records for those families in the UB Herbarium. An interesting fact is that although *Myrtaceae* is the second most recorded family in the dataset (with a total of 10,951 records), it was relatively overlooked by ‘*irwin,hs*’, having himself contributed with only 399 *Myrtaceae* records. The main *Myrtaceae* collector in the herbarium is *Jair E. Q. Faria* (*faria,jeq*), who apparently has a preference towards this family (it comprises 31.0% of his entire set of records). *Carolyn E. B. Proença* (*proenca,ceb*) is another key *Myrtaceae* collector, although she also seems interested, to the same extent, in families *Fabaceae* and *Asteraceae*. Moreover, Fig. 1 also makes it easy to detect collectors who exclusively (or almost exclusively) collect each family, as it is the case of *Vanessa G. Staggmeier* (*staggmeier,vg*) for *Myrtaceae* and *Regina C. Oliveira* (*oliveira,rc*) for *Poaceae*, for instance.

Community (*i*) in Fig. 1 represents a large part of the collectors from *Cryptogams Lab*, together with the taxa they are typically most interested in. This lab is part of the University of *Brasília* Department of Botany, having *Paulo Eduardo A. S. Câmara* (*camara,peas*), *Micheline C. Silva* (*carvalhosilva,m*), and *Maria das Graças M. de Souza* (*souza,mgm*) as the principal investigators. The first two researchers, included in community (*i*), are mostly interested in bryophytes (mosses and liverworts), mainly those from families *Sematophyllaceae*, *Hypnaceae*, and *Dicranaceae*. *Micheline C. Silva* also shows interest towards *Piperaceae*, a family of flowering plants that is also fairly recorded by some collectors from the network core. Therefore, *Piperaceae* is an important node connecting community (*i*) to the network core, as it intermediates many paths between collectors from both network regions. Although she is one of the principal investigators of the *Cryptogams Lab*, *‘souza,mgm’* was placed in community (*iii*), instead of (*i*), due to her taxonomic interest towards algae, a taxonomic group that is overlooked by the vast majority of collectors in the UB Herbarium, including bryophytes collectors. She is mostly interested in families *Eunotiaceae*, *Naviculaceae*, and *Pinnulariaceae*, which compose a group of algae known as diatoms.

### The UB Collector coWorking Network

As the UB CWN was built based on the same set of records as the SCN model explored in the previous section, the number of nodes is 6,768, equivalent to the number of collectors nodes in the SCN model. A total of 10,391 edges represent collaborative ties between collectors. The average degree and density for the overall network are, respectively, 3.07 and 4.5 × 10^−4^.

#### Connected components

The UB CWN is composed of a a total 2,991 connected components, the largest of which (*i.e*., the giant component *c*_1_) contains 46% of all nodes in the network. Such a relatively low percentage of nodes in the giant component contrasts to most empirical scientific paper-publishing collaboration networks studied by Newman^29^, with giant components containing as much as 80% to 90% of all nodes. Moreover, only 318 of the connected components in the UB CWN (*c*_1_, *c*_2_, …, *c*_318_) are composed of collectors with at least one collaborative tie. The remaining 2,673 components (*c*_319_, *c*_320_, …, *c*_2991_) are all disconnected nodes (*i.e*. nodes with degree *k* = 0), which we refer to as *individualist* collectors. Individualist collectors are those who have never recorded specimens collaboratively—or at least they have not included the names of their collaborators in the records as authors—, and thus are considered to have no structural role in the collaboration network of collectors. They comprise 39.5% of all nodes in the network, and the fact that they lack connections impacts on the overall network density, making it relatively low. In fact, if we instead compute network density by only considering nodes from the giant component *c*_1_, we observe an increase in density from 4.5 × 10^−4^ to 1.95 × 10^−3^.

One important example of an individualist collector in the herbarium is ‘*leite,alta*’, with a total of 2,757 records, none of which recorded as made collaboratively. This comprises 18.14% of all records by individualist collectors. All other 2,672 individualist collectors have fewer than 400 records each.

#### How collaborative are collectors

By inspecting the team sizes of all records in the dataset, we find that the average team size for records in the UB dataset is 1.73, as a consequence of the fact that a large part of all records are non-collaborative, *i.e*. recorded by a single collector. The number of records as a function of team size seems to decay logarithmically. Collectors from the UB Herbarium vary substantially regarding their collaborativeness on fieldwork. Whereas few ones have collaborated with a large number of collectors throughout their careers (much more than the average 3.07), many of them hold very few collaborative ties. In fact, almost 40% of them are individualist collectors, having never co-authored a single record.

**Figure 2:**
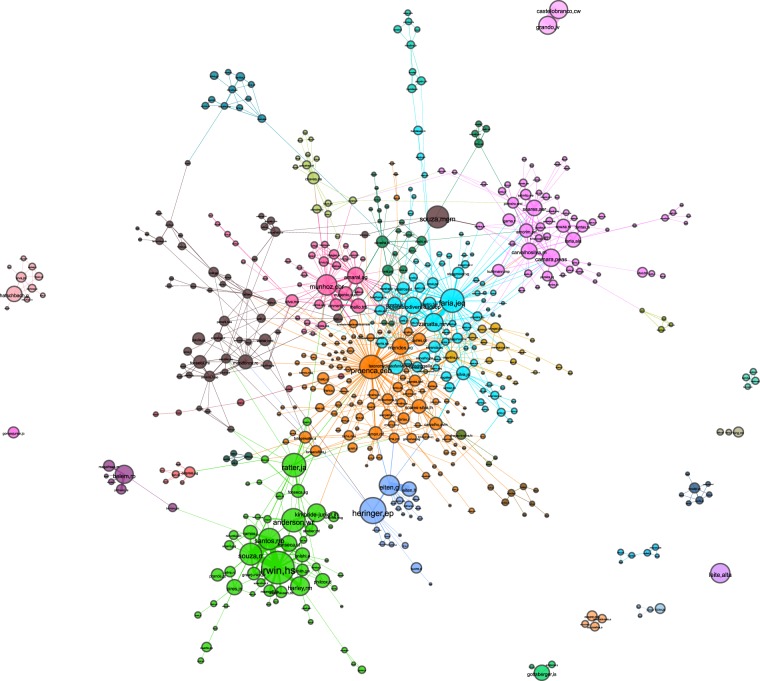
General aspect of the UB CWN. A total of 30 distinct coworking groups have been identified, and are differentiated by color. Node size is proportional to how often a collector appears on records, whilst the width of edges reflext their weight. This figure has been originally presented in the first author‘s MSc thesis^28^. For a better visualization experience refer to the interactive version of the graph (https://lncc-netsci.github.io/pedrocs/networks/ub_cwn).

The vertex *betweenness* centrality metric is also frequently used in social network analytics for ranking nodes that act as “bridges”, intermediating a considerable fraction of shortest paths between pairs of nodes in the network. We compute betweenness centrality of a node *v* by making ${c}_{B}(v)={\sum }_{s,t}\frac{\sigma (s,t| v)}{\sigma (s,t)}$, where *V* is the node set; *σ*(*s*, *t*) is the number of shortest paths between nodes *s*, *t* ∈ *V* (both *s* and *v* different from *v*); and *σ*(*s*, *t* ∈ *v*) is the number of shortest paths between *s* and *v* that are pass through *v*^30^. The 4 collectors with the highest betweenness centrality scores in the UB CWN are ‘*proenca,ceb*’ (0.38), ‘*faria,jeq*’ (0.18), ‘*mendes,vc*’ (0.14), and ‘*ratter,ja*’ (0.13). By inspecting Fig. 2, we verify that in fact all those collectors are in strategic positions. *Carolyn E. Proença* (*proenca,ceb*) is located at the center of the network, while ‘*mendes,vc*’ and ‘*faria,jeq*’ also interconnect nodes from many distinct communities. *James A. Ratter* (*ratter,ja*) is a very important node bridging a very relevant group (the green one, around ‘*irwin,hs*’) to the remainder of the network. Through the diversity of connections they had with other collectors, this top collectors from the betweenness perspective act as “bridges” connecting different communities of collectors.

#### Coworking groups

One important aspect that can be investigated from the topological structure of the UB CWN is the formation of communities of collectors who co-author specimen records, which we refer to as coworking groups. Nonetheless, visual inspection of the network structure does not allow us to fully identify the communities in this case. In order to detect such groups we applied the *Louvain* heuristic method for community detection^31^. The Louvain algorithm maximizes modularity scores in the network in successive steps, whereas the adopted modularity index is the one proposed by Newman^32^. The software we used for that is the python-louvain module for the NetworkX package^a^.^1^ The result is a partition of the network into modules (communities) within which nodes are more densely connected with each other than with external ones. Using this method, we detected a total of 30 distinct communities, 11 of which are detached from the giant component. For graph visualization, we first performed a filtering routine. We first filtered out weaker edges (those with hyperbolic weighting from Equation 3 lower than 10), which resulted in many islands, i.e. isolated components. We assigned scores to each island by summing up the weights of all nodes composing them. Islands with scores lower than 600 were omitted. The resulting graph has 545 nodes, 1,158 edges, and an average degree of 4.25 (Fig. 2). Among the communities, we identify the bryophytes research group, which includes ‘*camara,peas*’, ‘*carvalhosilva,m*’, and ‘*soares,aer*’. Collectors from this coworking group (colored in purple and located in the upper-right region of the giant component in Fig. 2) not only mostly collaborate with members from the same group in fieldwork, but they are also interested on recording the specific taxonomic group of bryophytes.

## Discussion

In this work, we introduced two network models that contribute towards a network-based conceptual framework for understanding the formation of biological collections as a result of the composition of collectors’ interests and activities. To this end, such network models represent the conceptual basis of a new approach for describing the assemblage of biological collections as a social process, driven by the taxonomic interests of contributor collectors as well as their social interactions. In this context, we provided methods for structuring species occurrence data from biological collections into two main classes of network models, each giving distinct perspectives on the recording behaviour of collectors. Species-Collector Networks (SCNs) model interest relations between collectors and taxa they record, whilst Collector coWorking Networks (CWNs) represent collaborative ties between collectors co-authoring records of specimens. As a case study, we demonstrated the use of our network models by exploring the species occurrence dataset of the University of Brasília (UB) Herbarium. Using the social network analytics framework^20,21^ as a theoretical foundation, we explored structural properties of the studied networks as well as investigated the formation of communities of collaboration and common interests. We also assessed the distinctiveness of collectors regarding their taxonomic interests and their collaborativity with others. Although in this study we specifically discuss SCNs and CWNs in the context of scientific biological collections, the same ideas here exposed can be also extended to other communities, such as those of nature observers (*e.g*. wildlife photographers, bird watchers) and citizen scientists.

We believe our network models provide the structural basis for a more realistic understanding on how collector and taxonomic biases arise in biological collections. As stated by Marin and Wellman^33^, network-based approaches allow analysts (*i*) to investigate the effects of interactions between individuals on shaping their own behaviours, rather than simply comparing static attributes of individuals within a population; and (*ii*) to investigate the formation of non-homogeneous communities, composed of individuals interacting with their groups at varying levels of commitment. We consider that these two aspects are particularly relevant in the context of biological collections.

First, collectors often start their careers being supervised by one or more experienced collectors. As it naturally happens in many social systems, the behaviour of individuals can be strongly influenced by others at more privileged positions, and this is likely to be the case for collector communities as well. A network-based approach would allow us to investigate, for instance, how the collecting behaviour and taxonomic interests of novice collectors are shaped by their association with more experienced ones. Moreover, depending on its position on the network, a collector can interact with multiple groups of collectors at different extents, thus assuming the role of influencer in some cases while being influenced in others. The influential power of a collector depends not only on the absolute number of connections it holds with others, but also on how strongly it intermediates other connections, how close it is to every other collector in the network, and how influential are its own connections. All these aspects can be assessed using well-known network centrality metrics (including degree, betweenness, closeness, and eigenvector centralities), and could be used for investigating which collectors are the most relevant for shaping the taxonomic composition of a biological collection from different perspectives.

Second, although collectors often define their own taxonomic interests and expertises in terms of natural or functional groups of organisms, those are not necessarily the groups that best split the interests of collectors in the dataset of a given collection. In addition, collectors (even the most specialized ones) are not restricted towards exclusively recording organisms of their expertises, nor they have uniform interest towards all of those organisms. In this context, SCNs provide the structure for discovering groupings of taxa that are better for characterizing and differentiating collectors based on their taxonomic interests (communities in the species projection); and for investigating associations of collectors with groups of taxa in a non-discrete manner, allowing collectors to be linked to taxa at multiple groups and at different intensities. In fact, while some groups of taxa are more specifically recorded by distinctive communities of specialized collectors, others are recorded by a wider range of collectors, with diverse taxonomic interests.

In this context, we believe the approach we introduce in this paper brings new perspectives on analysing data from biological collections based on social network analytics, including a broad range of potential further applications, given adequate and enough quality data is available as input. For instance, the knowledge extracted from the investigated networks can be used to plan better sampling campaigns. The studied methods can also be used to identify previously unrecognized biases or temporal trends in the culture of biological collecting. Some results may also be combined with new metrics to quantify, for instance, levels of “ignorance” in biological collections.^34^

The quality of our network models, however, strongly depends on the quality of the species occurrence dataset that is used to build them, more specifically on the fields containing the names of the collectors (*recordedBy*, in TDWG standards) and the taxonomic identity of the specimen (*scientificName*, in TDWG standards) of each record. We decided to only use the dataset from the UB in our case study because of its relatively high quality, specifically for the two aforementioned fields. In all other herbaria we examined, the collectors field (*recordedBy*) was particularly problematic. Our hypothesis is that the low quality of this field is associated with its low value for most uses of species occurrence data. While imprecise taxonomic determinations in the *scientificName* field would also lead to low quality networks, this field is critical for many other applications of occurrence data, and thus improving its quality has been extensively pursued by the biodiversity informatics community. The most common and impacting issues associated with the collectors field were: (*i*) using inconsistent delimiter characters for separating the names of each collector in a record, leading to many non-atomized names and consequently to the existence of nodes in the network that represent more than one collector; (*ii*) registering collectors names using inconsistent naming conventions, which makes it hard to systematically interpret what are the component parts of a name; (*iii*) using multiple name variations for a collector, leading to collectors being represented by more than one node in the resulting network; and (*iv*) only including the name of the first collector in records (and eventually aggregating all secondary collectors under the expression ‘*et al*.’), which is interpreted as an absence of collaborative ties and thus does not contribute for the formation of edges in CWNs. Constructing the network models from a low-quality dataset can therefore introduce several semantic imprecisions. We expect works as ours, which build upon the information about the collectors to enable new network-based perspectives on analysing data from biological collections, can motivate improved data curation to ease the construction of network models as those we investigate.

Our network models, as proposed in this work, also have a set of limitations, which should be addressed in the future. One important limitation of our network models is that they are static and non-spatialized (*i.e*., they are temporally and geographically invariant), and limited to representing relationships of a single type each. This implies that relationships modeled in both networks are assumed to occur irrespective of temporal and geographic dimensions, which is clearly limiting for the phenomena they model. As the careers of collectors have limited lifespans, they can only possibly collaborate with others if their activity periods overlap in time. In addition, both coworking (between collectors) and interest (involving a collector and a taxon) relationships derive from collecting events—each happening at a determined geographic location and at some point in time—, being thus temporally and spatially constrained. Indeed, cultural shifts among collectors and the possible existence of temporal trends in collector behaviour are interesting points to investigate. Therefore, incorporating these two dimensions to our models is also central for capturing network evolution in their structure. As in many other social systems, relationships in SCNs and CWNs change in time, as new ties are constantly formed while older ones are broken. It is reasonable to consider that collectors interact with distinct groups of people throughout their careers, assuming distinct roles in each relationship. For instance, we hypothesize that earlier in their careers, collectors are more likely to assume relationships and have their interests influenced by their academic supervisors or other collectors who are more experienced. On the other hand, relationships assumed by collectors later in their careers tend to be the opposite, as they assume the role of the more experienced collector (and thus, the influencer). Further, depending on the stage of a collector’s career and the groups of collectors he/she interacts at that moment, we might observe substantial shifts in his/her taxonomic interests (while changes in his/her taxonomic interests can also lead to collaborating with different groups). Other factors can also influence the patterns of recording activity of a collector, including oscillations in the availability of financial resources for field expeditions, changes in his/her residence location, and even phenological aspects of plant species of his/her interest.

As many applications using our network models would need to combine the perspectives of both SCNs and CWNs, we also recognize the importance of adopting an unifying model for seamlessly integrating the two networks into a single structure. The requirement for such a model is that it represents two types of connections (interest and coworking) and two distinct sets of nodes (collectors and species), besides incorporating the temporal and geographical dimensions into its structure. Although the concept of *multilayer networks* provides a solution for modeling dynamic complex systems with many aspects of connectivity, literature around this topic is still incipient, with many proposals though little consensus on the best way to represent them^35^. In this context, some proposals emerged lately to model such higher-order networks, such as MultiAspect Graphs (MAGs)^36,37^ or stream graphs and link streams^38^. Other alternatives for higher-order models of complex networked systems are also discussed in a recent paper by Lambiotte *et al*.^39^.

## Data availability

The *Python* package we developed during this study, *Caryocar*, is publicly available: https://github.com/pedrosiracusa/caryocar.

## Footnotes

1 ^a^https://python-louvain.readthedocs.io/en/latest/
